# Polypharmacy and Medication Self-Efficacy in Older Emergency Department Patients

**DOI:** 10.1093/geroni/igaf122.4175

**Published:** 2025-12-31

**Authors:** Inessa Cohen, Natalia Sifnugel, Ling Han, Daniella Meeker, Christopher Carpenter, William Hung, Debra Tomasino, Ula Hwang

**Affiliations:** Yale University, Yale University, Connecticut, United States; NYU Langone Medical Center, New York, New York, United States; Yale School of Medicine, New Haven, Connecticut, United States; Yale School of Medicine, Yale University, Connecticut, United States; Mayo Clinic, Rochester, Minnesota, United States; James J. Peters Department of Veterans Affairs Medical Center, New York, New York, United States; New York University, New York, New York, United States; NYU Grossman School of Medicine, New York, New York, United States

## Abstract

Over 50% of older adults in the US take ≥5 medications, with prevalence reaching 76.8% in the emergency department (ED) setting. However, little is known about their medication self-efficacy (the ability to understand and follow medication regimens), which may influence adherence and adverse drug events when new prescriptions are added during ED visits. Our objective was to determine whether polypharmacy and potentially inappropriate medication (PIM) are associated with self-efficacy. We performed a logistic regression analysis using 5-site multicenter ED survey data linked to electronic health records from patients aged ≥65 years with baseline ED visits during 2019-2021. The predictors were polypharmacy (≥5 medications) and PIM use (≥1 high-risk medication, GEMRx criteria) of prescriptions taken within 1 year of the ED visit. The outcome was low medication self-efficacy (< 11 points MUSE-taking score) assessed at the ED visit. Covariates included insurance, education, marital status, race/ethnicity, caregiver support, dementia status, and frailty. Among 921 patients, 249 (27.0%) had polypharmacy and 66 (7.2%) had PIM use. Low medication self-efficacy was reported by 76 (37.8%) with polypharmacy and 26 (12.9%) with PIM use versus 125 (62.2%) and 40 (5.6%) without. Both polypharmacy (aOR 6.12, 95% CI 2.05-18.22, p = 0.001) and PIM use (aOR 33.65, 95% CI 5.81-214.35, p < 0.001) were associated with greater adjusted odds of low medication self-efficacy. Frailty modified this association, with weaker effects in more frail patients (OR 5.51, 95% CI 2.57-11.8, p = 0.003). These findings suggest that the ED is an important setting to identify older adults who may struggle with medication use.

